# Factors associated with oral health knowledge, attitudes, and practices among legal guardians of preschool children in the Peruvian capital

**DOI:** 10.1186/s12889-025-22099-3

**Published:** 2025-03-17

**Authors:** Stephani Vargas-Santivañez, Marysela Ladera-Castañeda, Gissela Briceño-Vergel, Enrique Yarasca-Berrocal, Cinthia Hernández-Vergara, Jose Huamani-Echaccaya, César Cayo-Rojas

**Affiliations:** School of Stomatology, Universidad Privada San Juan Bautista, Lima and Ica, Peru

**Keywords:** Knowledge, Attitudes, Practices, Oral health, Legal guardians, Educational status, Socioeconomic factor

## Abstract

**Background:**

Legal guardians frequently serve as role models for their children. The habits they instill in their children may prove effective strategies for establishing healthy oral hygiene behaviors. The present study examined the factors associated with the level of oral health knowledge, attitudes, and practices among legal guardians of preschool children in the Peruvian capital. Furthermore, the correlation between knowledge, attitudes, and practices in oral health was assessed.

**Methods:**

This cross-sectional, analytical study evaluated 560 legal guardians of preschool children from 30 private educational institutions between July and December 2022. A validated 20-question questionnaire was employed to assess legal guardians’ knowledge, attitudes, and practices regarding oral health. Pearson’s chi-squared test and Fisher’s exact test were utilized for bivariate analysis. For multivariate analysis, a Poisson regression model with robust variance was applied using the adjusted prevalence ratio (APR). Statistical significance was set at *p* < 0.05.

**Results:**

A total of 82.3%, 76.2%, and 78.4% of legal guardians showed insufficient knowledge, unfavorable attitudes, and incorrect practices in oral health, respectively. The results indicated that legal guardians with high school and non-university higher education were 5.62 and 4.17 times, respectively, more likely to have insufficient oral health knowledge than those with university higher education. The same legal guardians were 6.18 and 5.02 times more likely to have an unfavorable attitude towards oral health than those with a university education (APR = 6.18, 95% CI 2.88–13.26 and APR = 5.02, 95% CI 2.32–10.90, respectively). Furthermore, these legal guardians were 4.35 and 3.08 times more likely to have incorrect oral health practices compared to those with university education (APR = 4.35; 95% CI: 2.39–7.90, and APR = 3.08; 95% CI: 1.66–5.69, respectively). On the other hand, legal guardians with a monthly family income of less than 270 USD were 14% and 15% less likely to have unfavorable attitudes and incorrect practices, respectively, compared to those with an income of 270 USD or more (APR = 0.86; 95% CI: 0.78–0.95, and APR = 0.85; 95% CI: 0.77–0.93, respectively). Finally, there was a moderate direct correlation between knowledge and attitudes (Rho = 0.56, 95% CI 0.51–0.62), knowledge and practices (Rho = 0.59, 95% CI 0.53–0.65), and attitudes and practices (Rho = 0.43, 95% CI 0.36–0.50).

**Conclusion:**

The majority of legal guardians had insufficient knowledge, unfavorable attitudes, and incorrect practices in oral health. High school and non-university higher education were risk factors for poor knowledge, unfavorable attitudes, and incorrect practices. Having a monthly family income of less than 270 USD was a protective factor for unfavorable attitudes and incorrect practices. Finally, a moderate direct correlation was identified between legal guardians’ oral health knowledge, attitudes, and practices.

**Supplementary Information:**

The online version contains supplementary material available at 10.1186/s12889-025-22099-3.

## Background

Oral diseases represent a significant public health concern due to their potential to negatively impact individuals during their growth and development. These conditions can cause a range of adverse effects, including pain, discomfort, loss of function, and a reduction in overall quality of life [1]. The World Health Organization (WHO) reports that diseases of the oral cavity affect approximately 3.58 billion individuals, with dental caries being the most prevalent condition. Estimates suggest that 486 million children suffer from caries in their deciduous teeth, and the financial burden of treating this disease hinders many countries from implementing effective measures to prevent serious complications [2].

A report from the Peruvian Ministry of Health (MINSA) indicates that 85.6% of preschool children between the ages of 3 and 15 have dental caries. This suggests that dental caries affects nine out of ten preschool children. Similarly, 52.5% of students between the ages of 10 and 15 suffer from periodontal disease [3]. Accordingly, the MINSA advises parents to assume an active role in their children’s oral care, promoting healthy habits and regular dental visits, with the objective of ensuring optimal oral health and quality of life [4].

The Regional Directorate of Education in the Peruvian capital hosts the Educational Management Unit (UGEL) No. 1. The goal of the above organization is to provide the best educational service possible by providing comprehensive training, identity development, and boosting students’ self-esteem in early childhood, primary, and secondary schools [5, 6] spread across ten districts in the southern region of Lima [6]. According to reports from the Lima Sur Integrated Health Network Directorate (DIRIS-LS) [7], dental caries represents the fourth leading cause of general morbidity in outpatient visits, accounting for 5.2% of dental care in these districts. Furthermore, pulp and periapical tissue diseases represent 1.9% of these visits [8]. This situation indicates that oral health conditions among Peruvian children have remained relatively unchanged in recent years. Therefore, this research is important, as, despite advances in technology and health care, a high prevalence of oral diseases persists at this stage of life, underscoring the need to address this issue as a priority [9–11].

In this context, legal guardians play a crucial role in promoting oral hygiene habits, especially in preschool children, who depend on adult supervision to maintain adequate oral health [12]. It is important to find out what legal guardians know, how they feel, and what they do because they have a big impact on both preventing and treating oral diseases. It has also been shown that habits formed in childhood are more likely to be kept into adulthood. This makes it even more important to use educational and awareness-raising strategies to reinforce their role in children’s dental care [1, 13, 14].

Some studies have indicated that factors influence the oral health knowledge, attitudes, and practices of legal guardians. In India, mothers with basic school education were reported to have limited knowledge about oral disease preventive measures, the importance of tooth brushing, and dental visits, in contrast to mothers with higher education [13]. In Malaysia, it was reported that the older the age of the parents, the higher the mean oral health knowledge and attitude scores, and it was noted that parents with low income had a higher mean knowledge score than those with high income, and mothers were reported to have higher mean scores for oral health knowledge, attitudes, and practices compared to fathers [15]. Finally, Kuwait reported that parents with a university degree scored higher on oral health practices [16].

The present study examined the factors associated with the level of oral health knowledge, attitudes, and practices among legal guardians of preschool children in the Peruvian capital. Furthermore, the correlation between knowledge, attitudes, and practices in oral health was assessed. One null hypothesis was that there are no factors significantly associated with oral health knowledge, attitudes, and practices among legal guardians. The other null hypothesis was that there was no correlation between knowledge, attitudes, and practices in oral health among legal guardians.

## Methods

### Study design

This analytical and cross-sectional study was conducted with legal guardians whose preschool children were enrolled in thirty private educational institutions (Anglicana, Arenitas del Mar, Belisario Suarez, Caritas Graciosas de Dios, Cooperativa Nuevo Milenio, El Mundo de los Geniecitos, Euclides, General La Mar, Gotitas de Amor, Gracia Divina, Hogar del Niño Jesús, Houston, James Thompson, Jardín Mi Hogar, Jesús es mi Rey, John Dalton, Jireh, José Encinas Franco, José Maria Eguren, Marianito, Mi Pequeña Estrella, Mi San Martincito, Niño Belén, Niños de Villa, Ricardo Palma, San Antonio de Padua, San Juan Bautista de La Salle, Santiago Apóstol, Triunfo de Cristo, and Virgen del Carmen) in the Education Management Unit (UGEL) No. 1, located in the south of the Peruvian capital and comprising 10 districts (Lurín, Pachacamac, Pucusana, Punta Hermosa, Punta Negra, San Bartolo, San Juan de Miraflores, Santa María del Mar, Villa El Salvador, and Villa María del Triunfo). The study was conducted from July to December 2022, in accordance with the STROBE guidelines [17].

### Population and participant selection

The total population comprised 600 legal guardians (the authorized representative of the preschooler to the school, usually the parents or the person who has legal guardianship of the preschooler). The minimum sample size was 235 participants, calculated using the statistical software Epidat 4.2 with a formula for estimating a proportion in a finite population. The formula considered a significance level of 0.05, an estimation error of 5%, and an expected proportion of 50%. However, to reduce sampling error and to obtain more precise estimates of population parameters, it was decided to include the entire study population, resulting in *N* = 560 participants according to the eligibility criteria.

**Inclusion criteria**

- Legal guardians who voluntarily participated in the study and provided informed consent.
- Legal guardian of a preschool enrolled in one of the thirty private educational institutions belonging to UGEL No. 1.

**Exclusion criteria**

- Legal guardians who did not complete the questionnaire.

### Variables

The dependent variables considered were knowledge (insufficient or sufficient), attitudes (unfavorable or favorable), and practices (incorrect or correct) in oral health. The independent variables were monthly family income [13, 15] and educational level [1, 13]. The adjustment variables were age [1, 15], and gender [15]. The age group was divided into three groups according to the Stanones rule, which considers the mean and standard deviation in the calculation: [mean (x̄) ± 0.75 (standard deviation)]. In addition, the categorization of monthly family income was based on the Peruvian minimum living wage, equivalent to USD 270.

### Instrument application

An oral health instrument was improved and validated [18] [See supplementary material]. Three expert judges, one pediatric dentistry researcher and two dental researchers with more than 15 years of experience, evaluated its content and validated its pertinence, objectivity, relevance, timeliness, sufficiency, clarity, and methodology, resulting in an acceptable Aiken’s V (V = 0.83; 95% CI: 0.79–0.87).

The principal component factor analysis with Varimax rotation identified three dimensions: D1 (oral health knowledge) (K1-K8), D2 (oral health attitudes) (A1-A6), and D3 (oral health practices) (P1-P6). The item-item correlation determinant was *p* < 0.001, Bartlett’s test of sphericity was *p* < 0.001, and the Kaiser-Mayer-Olkin (KMO) measure was 0.827. All values were acceptable [19].

The 20-item questionnaire was divided into three dimensions:

1. Knowledge about oral health, which consisted of 8 items with the following answers: true, false and don’t know. Correct answers were scored with 1 point and incorrect answers were scored with 0 points, including ‘Don’t know’. If the participant obtained ≤ 5 points, it was considered “insufficient knowledge”.
2. Attitudes about oral health, which consisted of 6 items with the following answers: agree (1 point), indifferent (0 points), and disagree (0 points). If the participant obtained ≤ 4, it was considered “unfavorable attitudes”.
3. Oral health practices, which consisted of 6 items with the following answers: yes and no. We scored 1 point for correct answers and 0 points for incorrect ones. If the participant obtained ≤ 2 points, it was considered “incorrect practices”.

The cut-off points for the total scores of oral health knowledge, attitudes, and practices were calculated based on Stanones’ rule. Livingston’s K^2^ coefficient validated the precision of these cut-off points, yielding acceptable results of 0.79 for knowledge, 0.69 for attitudes, and 0.85 for practices [20].

The Cronbach’s alpha test was used to find out how consistent the whole instrument was. It gave an overall score of 0.86 (95% CI: 0.84–0.87) and scores of 0.68 (0.95% CI: 0.64–0.72) for knowledge, 0.67 (0.95% CI: 0.63–0.71) for attitudes, and 0.82 (0.95% CI: 0.79–0.84) for practices, which are all good reliability values. We surveyed 30 randomly selected legal guardians over a period of 10 days at two different times to assess the reproducibility of the instrument, altering the order of the questions to avoid recall bias [21]. We then correlated the total scores with Spearman’s test (Rho), as neither score showed a normal distribution. The Rho yielded a value of 0.90 (95% CI: 0.80–0.95), which was considered acceptable.

### Procedure

The questionnaire was made and shared synchronously between July 10 and December 20, 2022, to a legal guardian of each preschool enrolled in one of the thirty selected educational institutions of UGEL No. 1, through the virtual platform Google Classroom^®^. This was during the COVID-19 pandemic in the final stages of the health emergency. The legal guardian had access thanks to the classroom teacher’s assistance, with the prior authorization of the educational institution’s director. The informed consent form was on the first page of the virtual questionnaire, along with the contact details of the principal investigator and the institutional ethics committee. Upon acceptance, the participant received immediate access to the questionnaire’s questions and instructions for completion. The link to the questionnaire was shared via the Zoom^®^ platform within the first 20 min of class. All participants were free to decline the questionnaire if they did not wish to complete it during the course of the survey. Only the researchers had access to the data, and no personal details of the legal guardians, such as telephone number, name, and address, were required. To identify duplicates, we only asked them to code their name and age (e.g., SVS25). Additionally, we configured the virtual survey to limit participation to one per associated email to minimize duplication. Upon completion of the investigation, the principal investigator sent the results to the legal guardians who requested them via email.

### Statistical analysis

The data collected through the Google Classroom^®^ virtual platform was stored in a Microsoft^®^ Excel 2019 spreadsheet. The data were then imported into the Statistical Package for the Social Sciences (SPSS) version 28.0. Qualitative variables were described using absolute and relative frequencies, while quantitative variables were described using measures of central tendency and dispersion. Bivariable analysis was conducted using Pearson’s chi-square test, while Fisher’s exact test was employed for expected values less than 5. Spearman’s Rho was used toestablish the correlation of scores between knowledge, attitudes, and practices on oral health of legal guardians. Multivariable analysis was performed using a Poisson regression model with robust variance and an adjusted prevalence ratio (APR). All statistical analyses were set at a significance level of *p* < 0.05.

### Ethical aspects

All legal guardians gave their informed consent voluntarily. Additionally, this study was approved on February 5, 2022, by the Institutional Research Ethics Committee of the Universidad Privada San Juan Bautista with resolution No. 179-2022-CIEI-UPSJB, since it respected the bioethical principles of confidentiality, freedom, respect, and nonmaleficence set forth in the Declaration of Helsinki [22].

## Results

**Fig. 1:**
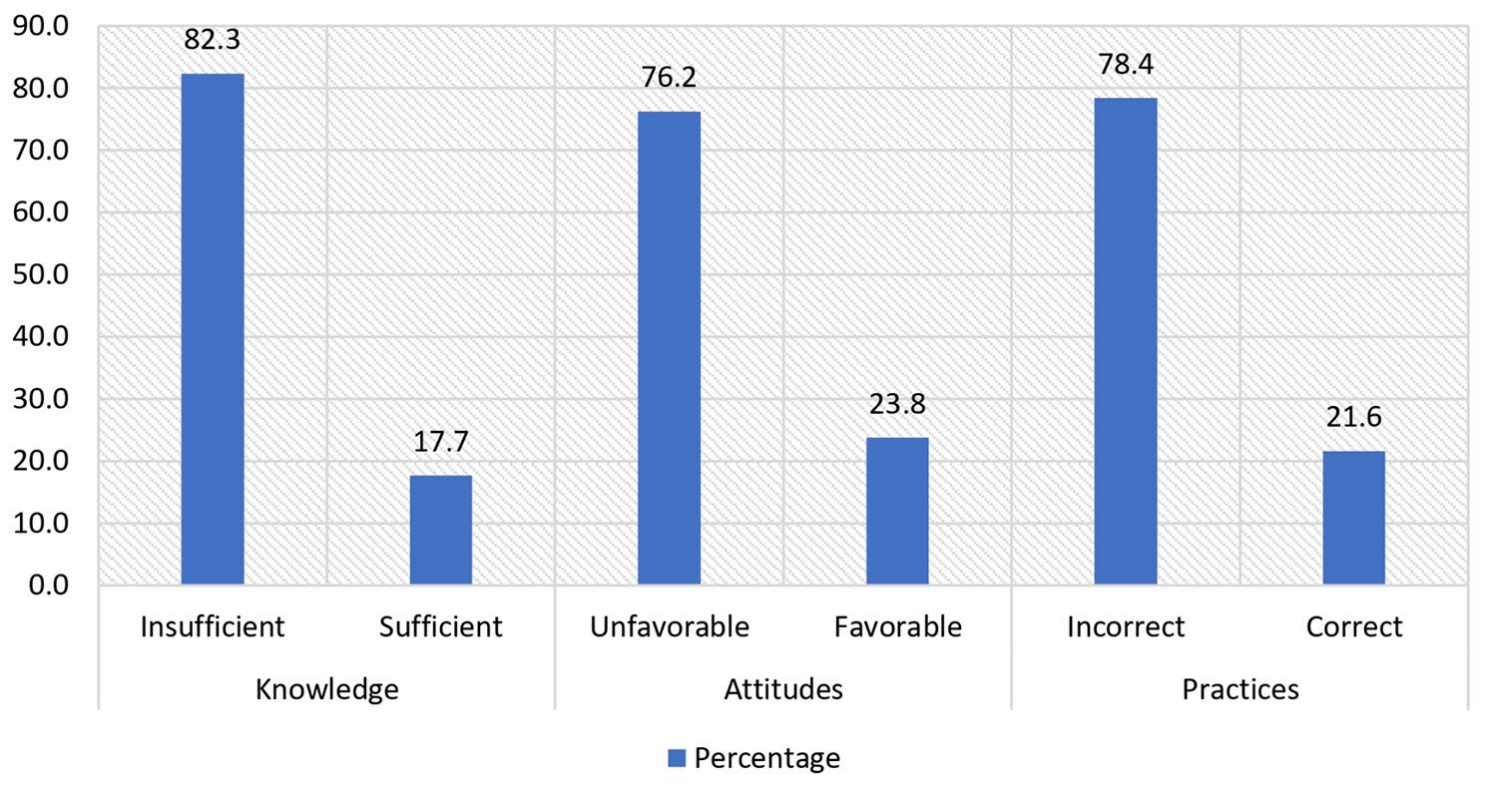
Relative frequencies of oral health knowledge, attitudes, and practices on oral health of legal guardians of preschool children in thirty educational institutions

The average age of the participants was 26.7 ± 2.9 years, of which 48.6% were between 25 and 28 years old. Of the total, 98.4% were women, and 70% of the respondents had a monthly family income of more than the minimum living wage, i.e., 270 USD or more. 77% of the legal guardians surveyed had only completed high school **[**Table 1**].** Finally, 82.3% (95% CI: 79.1–85.5%), 76.2% (95% CI: 72.7–79.7%), and 78.4% (95% CI: 75.0–81.8%) of all participants exhibit insufficient knowledge, unfavorable attitudes, and incorrect practices in oral health, respectively **[**Fig. 1**]**.

**Table 1:** Sociodemographic characteristics of legal guardians of preschool children in Thirty educational institutions

Variable	Category	Frequency	Percentage
**Age group**	20 to 24 years	133	23.8
	25 to 28 years	272	48.6
	29 to 35 years	155	27.7
**Gender**	Female	551	98.4
	Male	9	1.6
**Monthly family income**	< 270 USD	168	30.0
	≥ 270 USD	392	70.0
**Educational level**	High school	431	77.0
	Non-university higher education	82	14.6
	University higher education	47	8.4
**Age**	**Mean**	**Median**	**SD**
	26.7	27.0	2.9

*SD: Standard deviation*

Regarding oral health knowledge, it was observed that age group was significantly associated with all questions (*p* < 0.05), except K8 (is the concentration of fluoride in adult toothpaste the same as in children’s toothpaste?) (*p* = 1.000). Gender was significantly associated with all questions (*p* < 0.05), except K2 (can a baby’s first tooth appear as early as six months of age?) and K8 (*p* = 0.171 and *p* = 1.000; respectively). Monthly family income was significantly associated with all questions (*p* < 0.05), except K6 (does fluoride in toothpaste strengthen teeth and prevent dental caries?) and K8 (*p* = 0.087 and *p* = 0.300, respectively). Finally, educational level was associated with all questions (*p* < 0.05), except for K8 (*p* = 0.230) **[**Table 2**]**.

**Table 2:** Oral health knowledge associated with sociodemographic variables of legal guardians of preschool children in Thirty educational institutions

Question	Incorrect**	Correct	Age group	Gender	Monthly family income	Educational level
	f (%)	f (%)	p*	p*	p*	p*
**K1.** Is dental caries a stain that appears on the teeth due to the presence of microorganisms, sugar consumption, and a lack of hygiene?	186 (33.2)	374 (66.8)	0.006*	0.033*	< 0.001*	< 0.001*
**K2.** Can a baby’s first tooth appear as early as six months of age?	196 (35.0)	364 (65.0)	0.048*	0.171	< 0.001*	< 0.001*
**K3.** Are all baby teeth complete in the mouth by the age of two?	364 (65.0)	196 (35.0)	0.003*	0.001*	< 0.001*	< 0.001*
**K4.** Fats are the main type of food that can cause dental caries?	294 (52.5)	266 (47.5)	< 0.001*	0.016*	< 0.001*	< 0.001*
**K5.** Should tooth brushing start when the first tooth emerges?	275 (49.1)	285 (50.9)	0.009*	0.038*	< 0.001*	< 0.001*
**K6.** Does fluoride in toothpaste strengthen teeth and prevent dental caries?	333 (59.5)	227 (40.5)	0.027*	< 0.001*	0.087	< 0.001*
**K7.** Is it necessary to cure dental caries in baby teeth?	283 (50.5)	277 (49.5)	0.003*	0.019*	< 0.001*	< 0.001*
**K8.** Is the concentration of fluoride in adult toothpaste the same as in children’s toothpaste?	559 (99.8)	1 (0.2)	1.000	1.000	0.300	0.230

* Based on Pearson’s chi-square (p < 0.05,* significant association). For expected values less than 5*, Fisher’s exact test was used. **If the participant ticked the option ‘Don’t know’,* then it was considered as ‘Incorrect’*

Regarding oral health attitudes, age group was significantly associated only with A3 (a child’s toothbrushing should be done at least twice a day) (*p* < 0.001). Gender was significantly associated with A3 and A5 (It is important for a child to visit the dentist before the age of two) (*p* < 0.001 and *p* < 0.001; respectively). A1 (a child’s teeth are susceptible to attack by dental caries microorganisms), A4 (toothpaste is important for preventing dental caries), and A6 (a preschool children should brush teeth with adult supervision) were all significantly associated with monthly family income (*p* = 0.005, *p* = 0.007, and *p* < 0.001, respectively). Finally, educational level was significantly associated with all questions (*p* < 0.05) [Table 3].

**Table 3:** Oral health attitudes associated with sociodemographic variables of legal guardians of preschool children in Thirty educational institutions

Question	Unfavorable	Favorable	Age group	Gender	Monthly family income	Educational level
	f (%)	f (%)	p*	p*	p*	p*
**A1.** A child’s teeth are susceptible to attack by dental caries microorganisms.	133 (23.8)	427 (76.3)	0.207	0.124	0.005*	< 0.001*
**A2.** Good oral hygiene and a healthy diet can prevent dental caries.	129 (23.0)	431 (77.0)	0.171	0.127	0.092	0.003*
**A3.** A child’s toothbrushing should be done at least twice a day.	379 (67.7)	181 (32.3)	< 0.001*	< 0.001*	0.261	< 0.001*
**A4.** Toothpaste is important for preventing dental caries.	228 (40.7)	332 (59.3)	0.159	0.090	0.007*	< 0.001*
**A5.** It is important for a child to visit the dentist before the age of two.	350 (62.5)	210 (37.5)	0.097	< 0.001*	0.568	< 0.001*
**A6.** A preschool children should brush teeth with adult supervision.	150 (26.8)	410 (73.2)	0.834	0.456	< 0.001*	< 0.001*

* Based on Pearson’s chi-square (p < 0.05,* significant association). For expected values less than 5*,* Fisher’s exact test was used*

Regarding oral health practices, the age group had a significant correlation with P1 (Do you blow food to cool or taste it before giving it to your child?) P4 (Is the amount of toothpaste you use for brushing your child’s teeth the full length of the toothbrush bristles?) and P5 (Did you use a bottle with any sweet liquid when your child was an infant?) (*p* < 0.001, *p* < 0.001, and *p* = 0.023, respectively). Gender was significantly associated with all items (*p* < 0.05), except P1 and P2 (how often do you give sweet foods and juices to your child?) (*p* = 0.300 and *p* = 159, respectively). Monthly family income was only significantly associated with P6 (Should the child start dental visits before the first year of life?) (*p* < 0.001). Finally, educational level was significantly associated with all items (*p* < 0.05) [Table 4].

**Table 4:** Oral health practices associated with sociodemographic variables of legal guardians of preschool children in Thirty educational institutions

Question	Incorrect	Correct	Age group	Gender	Monthly family income	Educational level
	f (%)	f (%)	p*	p*	p*	p*
**P1.** Do you blow the food to cool or taste it before giving it to the child?	492 (87.9)	68 (12.1)	< 0.001*	0.300	0.214	< 0.001*
**P2.** Do you give your child sweet foods and juices on a regular basis?	471 (84.1)	89 (15.9)	0.087	0.159	0.496	< 0.001*
**P3.** Do you always clean your child’s mouth after eating?	434 (77.5)	126 (22.5)	0.067	< 0.001*	0.112	< 0.001*
**P4.** Does the amount of toothpaste you use for brushing your child’s teeth cover the entire length of the toothbrush bristles?	505 (90.2)	55 (9.8)	< 0.001*	< 0.001*	0.088	< 0.001*
**P5.** When your child was a baby, did you use a bottle with any sweet liquid?	467 (83.4)	93 (16.6)	0.023*	< 0.001*	0.131	< 0.001*
**P6.** Should the child begin dental visits before the first tooth erupts?	273 (48.8)	287 (51.3)	0.919	0.038*	< 0.001*	< 0.001*

* Based on Pearson’s chi-square (p < 0.05,* significant association). For expected values less than 5*,* Fisher’s exact test was used*

Based on the scores, there was a moderate direct correlation between knowledge and attitudes (Rho = 0.56; 95% CI: 0.51–0.62), knowledge and practices (Rho = 0.59; 95% CI: 0.53–0.65), and attitudes and practices (Rho = 0.43; 95% CI: 0.36–0.50); all three were significant (*p* < 0.001, *p* < 0.001, and *p* < 0.001) [Table 5].

**Table 5:** Correlation between knowledge, attitudes, and practices on oral health of legal guardians of preschool children in Thirty educational institutions

Variables	Rho	95% CI		p*
		LL	UL	
**Knowledge vs. Attitudes**	0.567	0.506	0.622	< 0.001*
**Knowledge vs. Practices**	0.592	0.534	0.645	< 0.001*
**Attitudes vs. Practices**	0.434	0.362	0.501	< 0.001*

The bivariable analysis of sociodemographic factors with regard to oral health knowledge, attitudes and practices revealed a statistically significant association between age group and knowledge (*p* < 0.001) and attitudes (*p* = 0.001). Similarly, gender was found to be associated with knowledge (*p* < 0.001) and attitudes (*p* < 0.001). However, no statistically significant association was observed between monthly family income and knowledge, attitudes, or practices (*p* > 0.05). Conversely, a significant association was found between educational level and knowledge, attitudes, and practices (*p* < 0.001, *p* < 0.001, and *p* < 0.001, respectively) **[**Table 6**].**

**Table 6:** Bivariable analysis of knowledge, attitudes, and practices in oral health associated with sociodemographic variables of legal guardians of preschool children

Variables	Categorías	Knowledge		p*	Attitudes		p*	Practices		p*
		Insufficient	Sufficient		Unfavorable	Favorable		Incorrect	Correct	
**Age group**	20 to 24 years	118 (88.7)	15 (11.3)	< 0.001*	104 (78.2)	29 (21.8)	0.001*	107 (80.5)	26 (19.5)	0.054
	25 to 28 years	233 (85.7)	39 (14.3)		221 (81.3)	51 (18.8)		221 (81.3)	51 (18.8)	
	29 to 35 years	110 (71.0)	45 (29.0)		102 (65.8)	53 (34.2)		111 (71.6)	44 (28.4)	
**Gender**	Female	460 (83.5)	91 (16.5)	< 0.001*	426 (77.3)	125 (22.7)	< 0.001*	438 (79.5)	113 (20.5)	< 0.001*
	Male	1 (11.1)	8 (88.9)		1 (11.1)	8 (88.9)		1 (11.1)	8 (8.9)	
**Monthly family income**	< 270 USD	144 (85.7)	24 (14.3)	0.168	125 (74.4)	43 (25.6)	0.502	126 (75.0)	42 (25.0)	0.202
	≥ 270 USD	317 (80.9)	75 (19.1)		302 (77.0)	90 (23.0)		313 (79.8)	79 (20.2)	
**Educational level**	High school	398 (92.3)	33 (7.7)	< 0.001*	364 (84.5)	67 (15.5)	< 0.001*	378 (87.7)	53 (12.3)	< 0.001*
	Non-university higher education	56 (68.3)	26 (31.7)		57 (69.5)	25 (30.5)		52 (63.4)	30 (36.6)	
	University higher education	7 (14.9)	40 (85.1)		6 (12.8)	41 (87.2)		9 (19.1)	38 (80.9)	

*Based on Pearson’s chi-square and for expected values less than 5,* Fisher’s exact test was calculated (p < 0.05*,* significant association)*

According to the Poisson regression model with robust variance and the adjusted prevalence ratio (APR), the dependent variables were insufficient knowledge (Yes [≤ 5 points] = 1 / No [> 5 points] = 0), unfavorable attitudes (Yes [≤ 4 points] = 1 / No [> 4 points] = 0), and incorrect practices (Yes [≤ 2 points] = 1 / No [> 2 points] = 0). Monthly family income and educational level were considered independent variables. Age group and gender were considered as adjustment variables. As a result, it was determined that legal guardians with high school and non-university higher education were 5.62 and 4.17 times more likely to have insufficient oral health knowledge compared to those with university higher education (APR = 5.62, 95% CI: 2.81–11.24, and APR = 4.17, 95% CI: 2.05–8.45, respectively). Those with high school and non-university higher education were 6.18 times and 5.02 times, respectively, more likely to have unfavorable attitudes about oral health compared to those with higher university education (APR = 6.18, 95% CI: 2.88–13.26 and APR = 5.02, 95% CI: 2.32–10.90; respectively). Finally, those with high school and non-university higher education were 4.35 times and 3.08 times, respectively, more likely to have incorrect oral health practices compared to those with university higher education (APR = 4.35, 95% CI: 2.39–7.90, and APR = 3.08, 95% CI: 1.66–5.69, respectively). On the other hand, legal guardians with a monthly family income of less than 270 USD were 14% and 15% less likely to have unfavorable attitudes and incorrect practices, respectively, compared to those with an income of 270 USD or more (APR = 0.86; 95% CI: 0.78–0.95, and APR = 0.85; 95% CI: 0.77–0.93, respectively) [Table 7].

**Table 7:** Multivariable analysis of knowledge, attitudes, and practices in oral health associated with sociodemographic variables of legal guardians of preschool children

Variable	Category	Knowledge					Attitudes					Practices			
		APR	95% CI		p*		APR	95% CI		p*		APR	95% CI		p*
			LL	UL				LL	UL				LL	UL	
**Age group [AV]**	20 to 24 years	1.03	0.94	1.13	0.501		1.00	0.88	1.13	0.988		0.95	0.85	1.06	0.337
	25 to 28 years	1.03	0.95	1.12	0.469		1.09	0.98	1.21	0.112		1.01	0.92	1.10	0.906
	29 to 35 years	*Ref.*					*Ref.*					*Ref.*			
**Gender [AV]**	Female	2.72	0.61	12.15	0.189		2.39	0.49	11.50	0.279		2.93	0.57	15.08	0.199
	Male	*Ref.*					*Ref.*					*Ref.*			
**Monthly family income**	< 270 USD	1.02	0.95	0.89	0.144		0.86	0.78	0.95	0.002*		0.85	0.77	0.93	< 0.001*
	≥ 270 USD	*Ref.*					*Ref.*					*Ref.*			
**Educational level**	High school	*5.62*	2.81	11.24	< 0.001*		6.18	2.88	13.26	< 0.001*		4.35	2.39	7.90	< 0.001*
	Non-university higher education	4.17	2.05	8.45	< 0.001*		5.02	2.32	10.90	< 0.001*		3.08	1.66	5.69	< 0.001*
	University higher education	*Ref.*					*Ref.*					*Ref.*			

*Adjusted multiple regression model (*p < 0.05,* significant association)*,* APR: Adjusted prevalence ratio under Poisson regression model with robust variance; 95% CI: 95% Confidence Interval; LL: Lower Limit; UL: Upper Limit. Age group and gender were the adjustment variables [AV] in each model*

## Discussion

The high prevalence of oral diseases makes it necessary to promote and prevent oral health from early childhood [1]. It is important to create awareness among legal guardians about children’s dental needs in order to reduce future oral health problems [1, 23], since these guardians are role models for their children [24]. In addition, healthy habits that children imitate or learn from their legal guardians during childhood can be effective strategies for establishing positive oral hygiene behaviors. Therefore, it is crucial to evaluate the oral health knowledge, attitudes, and practices of legal guardians to pinpoint areas that require enhancement for their children’s oral health [13, 25]. The present study examined the factors associated with the level of oral health knowledge, attitudes, and practices among legal guardians of preschool children in the Peruvian capital. Furthermore, the correlation between knowledge, attitudes, and practices in oral health was assessed. Based on the results obtained, the null hypotheses were rejected.

The results obtained in the present study showed that 82.3% of the legal guardians had insufficient knowledge. These results differ from those reported by Victorio-Pérez et al. [26], who found that 21% of legal guardians of preschool children aged 3 to 5 years in an area of the Peruvian capital had insufficient knowledge. The difference might be because this group had been getting oral health education for years from dental students at the Universidad Peruana Cayetano Heredia and from professionals at nearby health centers. In the present study, the legal guardians surveyed did not have any oral health education in their districts [26]. Also, the results from the present study differ from those reported by Benghasheer and Saub [15], who found that 15% of parents of children in Malaysia had insufficient oral health knowledge. This is probably due to the fact that 77% of the guardians surveyed in the present study had secondary school education, unlike the study by Benghasheer and Saub [15], in which 63.8% of parents had higher education. The results obtained in the present study showed that legal guardians with high school and non-university higher education were 5.62 times and 4.17 times, respectively, more likely to have insufficient oral health knowledge than those with university higher education. These results are similar to the study by Gaspar et al. [1], who reported that mothers with no education, elementary school, and high school were 11 times, 6 times, and 5 times, respectively, more likely to have poor knowledge compared to mothers with university higher education. This could be due to the fact that legal guardians with higher levels of education have greater access to adequate oral health information, possibly making them aware of the need to visit the dentist frequently [1, 13, 27] and thus improving their ability to promote their children’s oral health [1, 13, 15, 23, 25, 28].

It is known that the fluoride concentration of toothpastes in children should be between 1000 ppm and 1450 ppm, and the choice of fluoride concentration in toothpaste depends on the age of the child and the risk of dental caries [29]. In the present study, it was observed that only 0.2% of legal guardians considered the statement that fluoride concentrations were the same in adult and children’s toothpastes to be false (which was considered correct), and only 40.5% considered it true (which was considered correct) that fluoride in toothpastes strengthens teeth and prevents caries. This may be because many toothpastes don’t include fluoride concentration on their packaging. Also, the media can create confusion by promoting the choice of toothpaste based on taste and brand, not fluoride concentration, in addition to not mentioning the benefits that this chemical element brings to the teeth [30, 31]. Legal guardians with both high and low monthly incomes may be in this situation, as 96.2% of Peruvian households possess at least one information and communication device for accessing oral health information [32].

Regarding attitudes, 76.2% of the legal guardians showed an unfavorable attitude, which does not agree with that reported by Benghasheer and Saub [15] and Nepaul and Mahomed [33], who found that 8.1% and 5.6% of the parents, respectively, had a negative attitude towards oral health. This disagreement is possible because most of the guardians surveyed in this study had insufficient knowledge, which influenced their unfavorable attitudes [15, 25]. Also, the results showed that legal guardians with high school and non-university higher education were 6.18 times and 5.02 times, respectively, more likely to have unfavorable attitudes about oral health than those with university higher education. The legal guardians with secondary and non-university higher education likely lack familiarity with concepts such as dental caries, tooth brushing, toothpaste use, and the significance of dental visits [15]. It’s important to consider that the cognitive aspect of a topic plays a significant role in determining the development of attitudes [34–36].

The attitude about whether a child should brush their teeth at least twice a day was significantly associated with gender and age group. This could be because women, who are the primary caregivers and modelers of their children’s behaviors, are more concerned about their children’s toothbrushing [37, 38] and have a better attitude about it than fathers, who are generally more involved in financial support [37]. In addition, some studies indicate that younger guardians have better knowledge about the use of toothbrushes and toothpaste [38, 39], due to a better command of technology and the digital world, making it easier for them to self-report the frequency of brushing [39].

Regarding practices, 78.4% of the legal guardians had incorrect practices, which is discrepant with Benghasheer and Saub [15], who found that 12.3% of preschool guardians had incorrect practices. This is possibly due to the fact that most of the legal guardians surveyed in the present study had insufficient knowledge and unfavorable attitudes, which may have influenced their incorrect oral health practices. Benghasheer and Saub [15] conducted a study where the majority of respondents demonstrated positive knowledge and attitudes, leading to improved oral health practices. The present study also evidenced that legal guardians with high school and non-university higher education were 4.35 times and 3.08 times, respectively, more likely to have incorrect oral health practices compared to those with university higher education. This could be due to the fact that legal guardians with university education are better informed on oral health issues and have found it easier to implement better oral hygiene habits, reduce consumption of sweets, and use dental services in their families [40, 41]. Several studies show that parents with a high level of education have significantly better knowledge, attitudes, and practices regarding oral health care for their children [41–45]. Another possible explanation could be that these legal guardians encounter favorable socioeconomic, sociocultural, and sociobehavioral determinants unique to their residential districts [8], which are crucial in transforming health knowledge into effective health practices [16].

A significant correlation was observed between age group and the practices of blowing food to cool it, tasting food before giving it to the child, using an excessive amount of toothpaste for tooth brushing, and using bottles with sweet liquid. These practices may be considered incorrect by younger parents, since they have better access to digital information through social networks and may have better knowledge in this regard [38, 39]. It is important to emphasize that the aforementioned practices can be considered incorrect because blowing food can transmit bacteria, which can remain in the oral cavity until tooth eruption begins. The consumption of sugary foods and drinks can create dysbiosis and cause dental caries [46, 47]. Similarly, experts recommend that children under 3 years old should use toothpaste the size of a grain of rice, and from 3 years old onwards, they should use toothpaste the size of a pea [29, 48, 49].

The results show that there was a moderate direct correlation between knowledge, attitudes, and practices in a significant way. This could indicate that the more insufficient the knowledge, the more unfavorable the attitude, and the more incorrect the legal guardians’ oral health practices. Attitudes are based on individual experiences, knowledge, information received, positive and negative beliefs, and opinions about given situations [50]. Likewise, attitudes influence favorable or unfavorable behaviors; that is, behavior is the expression of attitudes in actions [50, 51]. The lack of knowledge and attitude towards oral health could constitute one of the barriers to the implementation of correct oral health practices, which in turn could influence the habits that legal guardians instill in their children [26, 41].

Another finding was that legal guardians with a monthly family income of less than 270 USD were 14% and 15% less likely to have unfavorable attitudes and incorrect practices, respectively, compared to those with an income of 270 USD or more. This result was surprising because legal guardians with low incomes have been reported to be at higher risk of poor oral health attitudes and practices [52–54]. This discrepancy with existing literature may stem from the fact that low-income legal guardians are more likely to rely on public health programs and free preventive campaigns, such as fluoridation, oral hygiene education, and care provided at health posts or community centers. As a result, they may have greater access to educational resources and best practices compared to those who can afford private care but do not prioritize prevention. Furthermore, the awareness of the high cost of dental treatment might motivate some low-income guardians to be more proactive in maintaining their oral health, thereby preventing unnecessary expenses in the future. This could lead to the adoption of beneficial preventive behaviors, such as regular brushing, using mouthwash, and participating in free dental check-up campaigns.

The present study is relevant because, despite multiple advances in technology, a high prevalence of oral diseases in childhood still persists [15, 23]. Legal guardians play a crucial role in maintaining excellent oral health, particularly in preschool children, because at this age children cannot brush their teeth properly by themselves and ignore the importance of preserving them due to their poor manual skills and mental immaturity. Furthermore, during a period known as “primary socialization,” children under six years of age spend most of their time with their parents, absorbing their daily routines [25]. It is essential to identify whether parents have sufficient knowledge, favorable attitudes, and correct practices in order to take corrective actions and instill positive oral health habits in their children [13, 16, 25, 55–58].

Bivariable analysis indicated an association between age and sex with respect to legal guardians’ oral health knowledge and attitudes. However, analysis using a robust multivariable regression model revealed that these variables were not associated with knowledge and attitudes. This finding suggests that the bivariate association does not necessarily imply a causal relationship or influence. Therefore, it is recommended that future studies incorporate multivariable statistical analysis under regression models when assessing factors influencing the knowledge, attitudes, and practices of legal guardians of preschool children [59].

The present study encountered some limitations. Since Peru was still in the final stages of the health emergency at the time of the survey, it was not possible to evaluate legal guardians in person [60, 61]. Likewise, the cross-sectional design used did not allow us to observe the variation over time in knowledge, attitudes, and practices regarding oral health among legal guardians. The restrictive measures prevented us from comparing knowledge, attitudes, and practices with guardians from public institutions in rural areas [62, 63]. Finally, while these results may not apply to all legal guardians across the country, they serve as a foundation for future research that promotes oral health. We suggest developing similar studies that involve legal guardians from both public and private institutions across various regions of Peru.

Based on the findings, health facilities should implement preventive oral health programs in educational institutions, collaborating with dentists and teachers, to raise awareness among legal guardians and improve oral health care habits in children [58]. Likewise, it would be advisable for dentists to provide oral health counseling to legal guardians and their children in order to identify possible risk factors for developing oral diseases and, in turn, minimize misconceptions and doubts [64, 65]. Finally, educational interventions are needed to improve the knowledge, attitudes and practices of legal guardians on current oral health guidelines and recommendations. This will have a positive impact on a child’s quality of life [41, 66].

## Conclusion

The majority of legal guardians had insufficient knowledge, unfavorable attitudes, and incorrect practices in oral health. High school and non-university higher education were risk factors for poor knowledge, unfavorable attitudes, and incorrect practices. Having a monthly family income of less than 270 USD was a protective factor for unfavorable attitudes and incorrect practices. Finally, a moderate direct correlation was identified between legal guardians’ oral health knowledge, attitudes, and practices.

## Electronic supplementary material

Below is the link to the electronic supplementary material.

Supplementary Material 1

## Data Availability

All data analyzed during this study are available from the corresponding author on reasonable request (cesarcayorojas@gmail.com).

## Abbreviations

APR: Adjusted Prevalence Ratio
CI: Confidence interval
DIRIS-LS: Lima Sur Integrated Health Network Directorate
MINSA: Peruvian Ministry of Health
STROBE: STrengthening the Reporting of OBservational studies in Epidemiology
SPSS: Statistical Package for the Social Sciences
WHO: World Health Organization
