# Supplementary material for: Factors associated with oral health knowledge, attitudes, and practices among legal guardians of preschool children in the Peruvian capital
Source: BMC Public Health. 2025 Mar 17;25:1030. doi: 10.1186/s12889-025-22099-3 (PMC11916944; doi:10.1186/s12889-025-22099-3)
Supplement: Supplementary file 1 — Supplementary Material 1 [file 12889_2025_22099_MOESM1_ESM.docx]

Supplementary material: Oral health questionnaire

| Knowledge | True | False | Don't know |
| --- | --- | --- | --- |
| **K1.** Is dental caries a stain that appears on the teeth due to the presence of microorganisms, sugar consumption, and a lack of hygiene? |  |  |  |
| **K2.** Can a baby's first tooth appear as early as six months of age? |  |  |  |
| **K3.** Are all baby teeth complete in the mouth by the age of two? |  |  |  |
| **K4.** Fats are the main type of food that can cause dental caries? |  |  |  |
| **K5.** Should tooth brushing start when the first tooth emerges? |  |  |  |
| **K6.** Does fluoride in toothpaste strengthen teeth and prevent dental caries? |  |  |  |
| **K7.** Is it necessary to cure dental caries in baby teeth? |  |  |  |
| **K8.** Is the concentration of fluoride in adult toothpaste the same as in children's toothpaste? |  |  |  |
|  |  |  |  |
|  |  |  |  |
| Attitudes | Agree | Indifferent | Disagree |
| **A1.** A child's teeth are susceptible to attack by dental caries microorganisms. |  |  |  |
| **A2.** Good oral hygiene and a healthy diet can prevent dental caries. |  |  |  |
| **A3.** A child's toothbrushing should be done at least twice a day. |  |  |  |
| **A4.** Toothpaste is important for preventing dental caries. |  |  |  |
| **A5.** It is important for a child to visit the dentist before the age of two. |  |  |  |
| **A6.** A preschool children should brush teeth with adult supervision. |  |  |  |
|  |  |  |  |
|  |  |  |  |
| Practices | Yes | NO |  |
| **P1.** Do you blow the food to cool or taste it before giving it to the child? |  |  |  |
| **P2.** Do you give your child sweet foods and juices on a regular basis? |  |  |  |
| **P3.** Do you always clean your child's mouth after eating? |  |  |  |
| **P4.** Does the amount of toothpaste you use for brushing your child's teeth cover the entire length of the toothbrush bristles? |  |  |  |
| **P5.** When your child was a baby, did you use a bottle with any sweet liquid? |  |  |  |
| **P6.** Should the child begin dental visits before the first tooth erupts? |  |  |  |
